# Tear film assessment before and after phacoemulsification in patients with age-related cataracts

**DOI:** 10.1186/s12886-024-03542-2

**Published:** 2024-07-11

**Authors:** Yasmine Maher Shaaban, Bassem Fayez Aziz

**Affiliations:** 1 Department of Ophthalmology, Faculty of Medicine, Ain Shams University, Abbassia, Cairo, 11591 Egypt; 2 Ain Shams University Specialised Hospital, Clinics, and Ophthalmology Investigative Unit, Khalifa El-Maamoun, Heliopolis, Cairo Governorate, 11588 Egypt; 3 El Watany Specialized Eye Hospital, 211 El-Hegaz St, Heliopolis, Cairo, 11351 Egypt

**Keywords:** Phacoemulsification, Break-up time, Tear height, Meibomian glands, Lipid layer

## Abstract

**Background:**

The study aims to assess the tear film before and after phacoemulsification in patients with age-related cataracts.

**Methods:**

A prospective observational study of 41 age-related cataract patients undergoing phacoemulsification procedure. Tear Film Break-Up Time (TBUT), Tear Film Meniscus Height (TMH), Meibomian glands (MG), and Lipid Layer Thickness (LLT) were assessed by a non-invasive Dry Eye Diagnostic System. All measurements were taken preoperatively, one week, one month, and three months postoperatively. The Marginal homogeneity and The Cochran Q tests were used in the statistical analysis.

**Results:**

The value of Non-Invasive Break-Up Time (NITBUT) was statistically significantly lower at one week (7.15 ± 3.31), one month (7.61 ± 3.41), and three months (7.66 ± 3.36) postoperatively than preoperatively (10.71 ± 2.71), *p* < 0.001. The Non- Invasive Tear Meniscus Height (NITMH) was significantly lower at one week (0.18 ± 0.0), one month (0.20 ± 0.09), and three months (0.20 ± 0.09) postoperatively than preoperatively (0.30 ± 0.113) *p* < 0.001. By the first month, both (NITBUT) and (NITMH) improved significantly compared to the first post-operative week. There was no statistically significant difference between one month and three months. The (NITMH) improved to a healthy level of ≥ 0.2 mm by the first month through the third month. Both (NITBUT) and (NITMH) did not reach the baseline by the third month. The meibomian glands and the lipid layer thickness had the same preoperative grade distribution without changes.

**Conclusion:**

Phacoemulsification surgery can cause post-operative deterioration in the tear film, which starts within a week of the procedure, followed by gradual recovery over the next weeks and months. The phacoemulsification procedure mainly affects the tear break-up time and tear meniscus height. Both the lipid layer and meibomian glands are not affected.

## Background

Dry eye is an ocular surface disease affecting the tear film [1]. Dry eye disease (DED) causes ocular discomfort, fatigue, and visual disturbance that interferes with quality of life, including different aspects of physical, social, and psychological functioning, daily activities, and workplace productivity [2]. The causing factors include old age, female gender, autoimmune diseases, systemic hypertension, diabetes mellitus, and contact lens use [3]. Some drugs like anticholinergics, antidepressant drugs, oral contraceptives, and topical eye drops containing preservatives can also cause dry eye [4, 5]. Analgesics such as Aspirin and ibuprofen continue to be linked to dry eye. Both of these drugs are secreted into the tears, after which they can increase tear evaporation and affect tear film stability [6].

Systemic or topical ocular medications and preservatives used in topical ocular drugs may cause dry eye through the drug’s therapeutic action, ocular surface effects, or preservatives, and the effects probably are additive [7].

Phacoemulsification has become a major procedure in cataract surgery. Postoperative dry eye can occur due to decreased corneal sensation due to the incisions [8], postoperative use of antibiotic-steroid eye drops or ointment [9], post-operative ocular inflammation [10], and light exposure from the surgical microscope [11]. The risk factors for Dry eye Diseases (DED) after cataract surgery included age, female sex, systemic diseases, systemic medications, psychiatric conditions, preexisting DED, meibomian gland dysfunction, preservatives in eye drops, surgery techniques, and lifestyle [12].

Conventionally, tear film evaluation includes testing for Tear Break Up Time (TBUT) using sodium fluorescein eye drops and the slit lamp. Assessment of the amount of tears produced by the eye is done by placing a strip of filter paper inside the lower fornix of an anesthetized eye, Schirmer tear test 1 (STT1) [13]. Lissamine Green (LG), and Rose Bengal (RB) stains, are also used to diagnose ocular surface disease, and tear film quality [14]. It also includes interferometry for lipid layer evaluation [15], and contact meibography to evaluate the meibomian glands [16]. Interferometry is an optical technique used to quantify the lipid layer of the precorneal tear film and investigate the relationship between lipid layer thickness and tear film evaporation. Contact Meibography **is** performed to detect possible Meibomian gland dysfunction (MGD) and to monitor their correct functioning.

Recently, based on artificial intelligence (AI), a non-invasive dry eye diagnostic system has been introduced for analysis of the tear film automatically. The data are provided quantitatively without touching or parameter settings. No sterilization is required. It avoids the discomfort due to Schirmer’s paper and the irritation from fluorescein. It is convenient for old and irritable patients.

## Methods

This prospective observational study aimed to assess the tear film before and after phacoemulsification in patients with age-related cataracts. The study was conducted at Ain Shams University Hospitals and El Watany Specialized Eye Hospital, Cairo, Egypt in the period between February 2023 and December 2023, Written Informed consent was obtained from each patient.

### Inclusion criteria

Cases with age-related cataracts without dry eye who were subjects for phacoemulsification procedure.

### Exclusion criteria

Cases with possible preoperative dry eye were excluded from the study. Cases of keratoconus, pre-existing corneal pathology, post-LASIK cases, diabetes mellitus, hypertension, rheumatoid arthritis, systemic lupus erythematosus, severe dry eye, and pregnant women.

All patients were examined; visual acuity was measured with a Landolt chart. Refraction was done with the auto refractometer. Anterior segment examination was done by the slit lamp and the grade of the cataract was evaluated. The fundus was examined with an indirect ophthalmoscope.

All surgeries were performed under peribulbar local anesthesia. Using a Zeiss Lumera surgical microscope (Carl Zeiss Meditec AG, Jena, Germany), a 2.4 mm clear superior corneal incision was made. Peribulbar (PB) local anesthesia is preferred to retrobulbar (RB) anesthesia as it carries no risk of injury to the optic nerve besides its added safety in case of staphylomas. PB local anesthesia is preferred to topical anesthesia as it provides better ocular stability for anxious patients.

The anterior chamber was filled with a dispersive viscoelastic 2% hydroxypropyl methylcellulose (OcuCoat Bausch + Lomb, MN, USA). The dispersive viscoelastic 2% hydroxypropyl methylcellulose is protective of the corneal endothelium.

A continuous curvilinear 5 mm capsulorhexis, hydrodissection, and two side port incisions were made. The phacoemulsification procedure was done using the Alcon Infinity Phaco-emulsification machine (Alcon Laboratories, Inc. Fort Worth, Texas, USA). The nucleus was managed by stop-and-chop technique and the cortex was aspirated using bimanual irrigation/aspiration. The capsular bag was filled with 1% Na Hyaluronate (Optiflex, Ireland). A foldable monofocal posterior chamber Intra Ocular Lens ( Sensar Johnson & Johnson, CA, USA) was implanted in the capsular bag through an injector system. The viscoelastic material was aspirated. The incisions were closed with stromal hydration. Postoperatively, patients used topical antibiotics (Moxifloxacin 0.5%, Prednisolone 1%) six times a day for a week that tapered over the following four weeks.

The stop-and-shop technique is the mastered technique by the surgeon. The 1% Na Hyaluronate is a visco-cohesive material that maintains the anterior chamber and flattens the anterior capsule. The used IOL is a mono-focal hydrophobic lens. Antibiotic eye drops are used to protect against infection, and steroids are used to prevent post-operative inflammation.

A Dry Eye Diagnostic System (MediWorks, Shanghai Precision Instruments Co., Ltd), is a recently invented system based on AI, it was used as an alternative non-invasive quantitative method to assess the Non-Invasive Tear Breakup Time (NITBUT), the Non-Invasive Tear Meniscus Height (NITMH}, Meibomian Glands (MG), and Lipid Layer Thickness (LLT). The NITBUT was analyzed automatically and quantitatively. The first breakup time and the average breakup time were provided according to the following grades: Grade 0 Normal, first rupture time: 10 s, and average rupture time: 14 s. Grade 1 Warning, first rupture time: 6–9 s, average rupture time: 7–13 s. and Grade 2 Dry eye, first rupture time: 5 s, average rupture time: 7 s. The NITMH was measured automatically. The normal value is ≥ 0.2 mm. The MG was automatically identified and analyzed for loss and dysfunction according to the following grading: Grade 0, no loss of meibomian glands, Grade 1, area of loss is less than one-third of the total meibomian gland area, Grade 2, area of loss is between one-third and two-thirds, and Grade 3, area of loss is more than two-thirds. A white ring projection system measured the LLT. The thickness was scored using grades 1–4, Grade 1 < 30 nm, Grade 2 = 30–60 nm, Grade 3 = 60–80 nm, and Grade 4 > 80 nm.

Data were collected and analyzed preoperatively, at one week, one month, and three months postoperatively. Measurements were expressed in seconds for the NITBUT, mm for the NITMH, and grades for the MG and LLT.

### Statistics

The sample Size: Using the PASS 15 program for sample size calculation, setting power at 80%, and alpha error at 0.05, it is estimated that a sample size of 35 eyes will be needed to detect a medium effect size difference between tear film assessment parameters before and after phacoemulsification procedure (d = 0.5). Descriptive and analytical statistical data are provided. Quantitative data were submitted as mean ± standard deviation while qualitative variables were submitted as grades.

Testing the differences in the proportion of NITBUT, and categories at different time points was done using the Marginal Homogeneity test between each pair and adjusting the significance level by Bonferroni correction. The differences in the proportion of NITMH two categories at different time points were tested using the Cochran Q test with a post-hoc pairwise comparison with Bonferroni correction. Data management and analysis were completed by SPSS (version 24).

## Results

This study includes 41 eyes of 18 male (44%) and 23 female (56%) patients aged between 46 and 79 years (65.2 ± 8.1). The grade of cataracts was between N1 and N5, and the time of surgery varied between 8 and 16 min (12.1 ± 2.2).

The pre-operative NITBUT is statistically significantly different from NITBUT at each subsequent point of time with *p* < 0.001 (Tables 1 and 2).

**Table 1:** NITBUT and NITMH at different time points (Mean and Standard Deviation)

	Gradings	Mean ± SD
**Pre-Operative NITBUT**	G 0 :10 s, Healthy	10.71 ± 2.71
One Week Post-Operative NITBUT	G1: 7–9 s, Warning	7.15 ± 3.31
One Month Post-Operative NITBUT	G1: 7–9 s, Warning	7.61 ± 3.41
Three Months Post-Operative NITBUT	G1: 7–9 s, Warning	7.66 ± 3.36
**Pre- Operative NITMH**	Healthy	0.30 ± 0.11
One Week Post-Operative NITMH	Abnormal	0.18 ± 0.08
One Month Post-Operative NITMH	Healthy	0.20 ± 0.09
Three Months Post-Operative NITMH	Healthy	0.20 ± 0.09

**Abbreviations: SD**, standard deviation; **G**, Grade; **NITBUT**, Non-Invasive Tear

Break Up Time; **NITMH**, Non-Invasive Tear Meniscus Height

**Table 2:** Pairwise comparisons of NITBUT at different time points

	STD MH	Sig.	Adj. Sig.*
Pre-Operative vs. one week Post-Operative	5	**< 0.001**	**< 0.001**
Pre- Operative vs. one month Post-Operative	4.315	**< 0.001**	**< 0.001**
Pre- Operative vs. three months Post-Operative	4.315	**< 0.001**	**< 0.001**
One week vs. one month Post-Operative	-2.828	**0.005**	**0.03**
One week vs. three months Post-Operative	-2.828	**0.005**	**0.03**
One month vs. three months Post-Operative	0	1	1.000

*Bonferroni correction

**Abbreviations: NITBUT**, Non-Invasive Tear Break-Up Time; **STD MH**, Standardized Marginal Homogeneity; **Sig**, Significant; Adj. Sig.*, Adjusted Significance

**Fig. 1:**
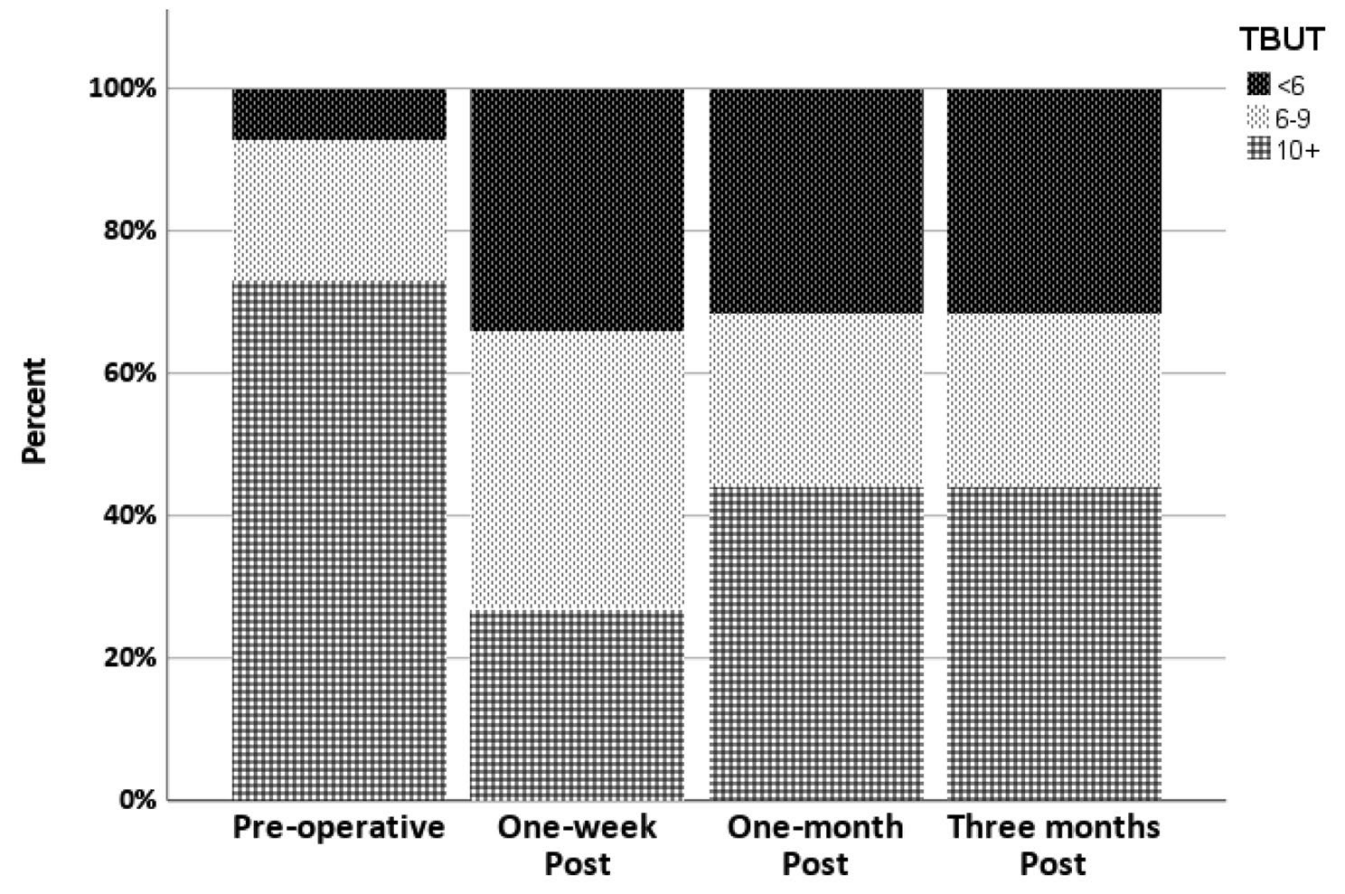
Stacked bar chart of NITBUT at different points of time

By the first week, the NITBUT was significantly lower. By the first month, it improved significantly compared to the first post-operative week. There was no statistically significant difference between one month and three months. The NITBUT did not reach the baseline by the third month (Fig. 1).

There was a statistical significance of the change in the proportion of healthy (NITMH) ≥ 0.2 mm between the four points of time with a test statistic of 30.067, df (3), and *p* < 0.001. The proportion of healthy (NITMH) was 92.7% and dropped significantly to 56.1%, 61%, and 61% in one week, one month, and three months postoperatively **(**Table 3**)**.

**Table 3:** NITMH at different time points (*N* = 41)

	≥ 0.2 mm	< 0.2 mm
Pre-operative NITMH	38 (92.7%)	3 (7.3%)
One Week NITMH	23 (56.1%)	18(43.9%)
One Month NITMH	25 (61%)	16 (39%)
Three Months NITMH	25 (61%)	16 (39%)

**Abbreviations: NITMH**, Non-Invasive Tear Meniscus Height

**Fig. 2:**
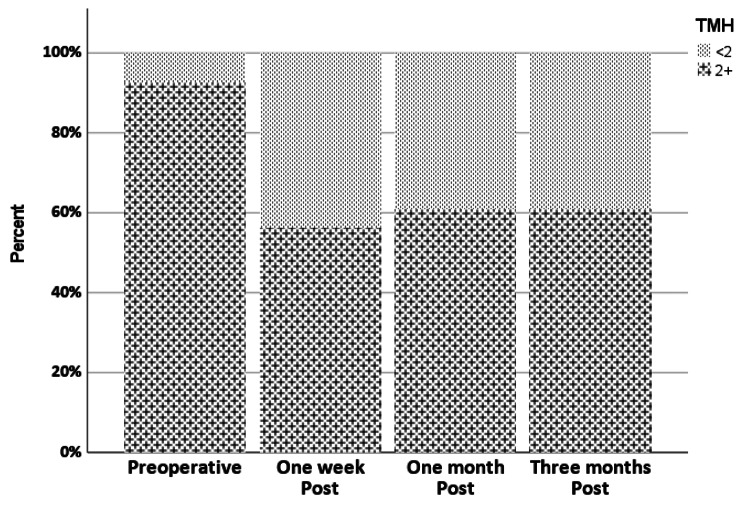
Stacked bar chart of TMH at different points in time

One week postoperatively, (NITMH) was significantly lower (0.18 ± 0.0) than the preoperative healthy level (0.30 ± 0.11). It improved to a healthy level (0.20 ± 0.09 ) in 61% of patients by the first month through the third month (Table 1). However, it did not reach the baseline value (0.30 mm) by the third month. The differences between one week, one month, and three months were insignificant (Tables 1 and 4; Fig. 2).

**Table 4:** Pairwise comparisons of NITMH at different time points

Sample 1-Sample 2	Test Statistic	STD. Error	STD. Test Statistic	Sig.	Adj.Sig.*
preoperative vs. one week	0.366	0.067	5.477	**< 0.001**	**< 0.001**
preoperative vs. one month	0.317	0.067	4.747	**< 0.001**	**< 0.001**
preoperative vs. three months	0.317	0.067	4.747	**< 0.001**	**< 0.001**
one week vs. one month	-0.049	0.067	-0.730	0.465	1.000
one week vs. three months	-0.049	0.067	-0.730	0.465	1.000
one month vs. three months	0.000	0.067	0.000	1.000	1.000

*Bonferroni correction

**Abbreviations: NITMH;** Non-Invasive Tear Meniscus Height, **STD**; Standardized, **Sig**, Significant.**Adj. Sig**.* adjusted significance

Regarding the meibomian glands (MG), the pre-operative measurements showed 8 cases (19.5%) in Grade 0, 22 cases (53.7%) in Grade 1, 6 cases (14.6%) in Grade 2, and 5 cases (12.2%) in Grade 3. The same grade distribution was found in the following three post-operative observations without change.

Regarding the lipid layer thickness, the pre-operative measurements showed 5 cases (12.2%) in Grade 1, 6 cases (14.6%) in Grade 2, 22 cases (53.7%) in Grade 3, and 8 cases (19.5%) in Grade 4. The same grade distribution was found in the following three post-operative observations without change.

## Discussion

This study used a noninvasive method to evaluate the tear film structure after phacoemulsification surgery at different intervals. Multiple studies have been done to evaluate the tear film in association with phacoemulsification, mostly using conventional evaluating methods.

In a study by Zamora et al. [10], 55 eyes of 55 different patients with no history of dry eye underwent standard phacoemulsification through a 2.75 mm-wide corneal incision. The TBUT, STT1, and TMH were measured. The OSDI score, fluorescein staining patterns, and photo documentation of the ocular surface before and during 1 day, 1 week, and 1 month postoperatively were recorded. In this study, phacoemulsification tends to induce short-term transitory ocular surface impairment manifesting as both signs and symptoms. All the dry eye tests were significantly deteriorating after surgery. Average break-up time values and the post-operative Schirmer value were significantly lower than the preoperative value (*p* < 0.001). Patients with altered preoperative values were more likely to develop ocular surface disease for a longer time.

A study by Kohli et al. [11] evaluated the signs and symptoms of dry eye after phacoemulsification; effects on the ocular surface using impression cytology; and associated risk factors. The study included 50 eyes (50 patients) with no dry eye signs or symptoms, who underwent clear corneal phacoemulsification for senile cataracts. The OSDI scoring, STT1, TBUT, TMH, corneal fluorescein staining, LG staining, and goblet cell density (GCD) with the help of impression cytology was done. Primary outcome measures included postoperative changes in the dry eye indices. Secondary outcome measures included correlation of the dry eye signs and symptoms with various risk factors. In this study, there was a transient sharp deterioration of dry eye in the immediate postoperative period after phacoemulsification. Severity improved gradually and recovery occurred by the end of the sixth week. Risk factors for deterioration include age, duration of exposure to microscope light, and effective phacoemulsification time. Kohli et al. recommended the use of minimum light exposure and ultrasound energy during the surgery.

Chao and LBS [17] studied the incidence of dry eye after phacoemulsification. Forty-nine (49) eyes of forty-four (44) Philippines patients diagnosed with age-related cataracts underwent clear cornea phacoemulsification and were enrolled in the study. The TBUT and STT1 were done before and after surgery. All values decreased at one week, and one month after the surgery. Recovery occurred by the 3rd month.

Cetinkaya et al. [18] assessed dry eye after phacoemulsification surgery. The study included 192 eyes of 96 patients with chronic dry eye syndrome and cataracts, who had undergone phacoemulsification surgery. Their mean age was (range 56–83) years. The mean postoperative 1st day, 1st week, and 1st-month TBUT values were significantly lower than preoperative TBUT values (*P* < 0.001), however 3rd month, 6th month, 1st year and 2nd-year values were not significantly different from preoperative values (*P* = 0.441, *P* = 0.078, *P* = 0.145, *P* = 0.125). The mean postoperative 1st day, 1st week, and 1st month STT1 values were significantly lower than preoperative STT1 values (*P* < 0.001), however 3rd month, 6th month, 1st year and 2nd-year values were not significantly different from preoperative values (*P* = 0.748, *P* = 0.439, *P* = 0.091, *P* = 0.214). Recovery occurred by the third month. Both the lipid and the meibomian glands were not affected by the study. This finding is in accordance with our study.

Yu et al. [19] compared the dry-eye signs and symptoms after femtosecond laser-assisted cataract surgery and conventional phacoemulsification. Patients were assessed using the ocular surface disease index (OSDI), subjective symptom questionnaire, Keratograph 4 corneal topography, STT1, and fluorescein staining preoperatively and postoperatively at 1 day, 1 week, and 1 month. Most patients developed dry eye postoperatively. Subjective symptoms and fluorescein staining scores elevated from baseline, TBUT and STT1 values decreased postoperatively, which peaked at 1 week and did not return to baseline within 1 month. There were no significant differences between the 2 groups (all *P* > 0.05) except for a higher fluorescein staining score in the femtosecond group at 1 day (*P* = 0.001), 1 week (*P* = 0.047), and 1 month (*P* = 0.025). The OSDI score and subjective symptoms were greater in the laser group at 1 week (*P* = 0.014 and *P* = 0.016, respectively). Subgroup analysis showed obvious worsening by fluorescein staining at 1 day (*P* = 0.016) and 1 month (*P* = 0.009) in preoperative dry-eye patients. Both methods worsened dry eye postoperatively. Femtosecond-assisted surgery had a higher risk for staining and dry-eye symptoms. Patients with preexisting dry eye who had femtosecond-assisted surgery had more severe ocular surface staining than those having conventional surgery.

Cung et al. [20] studied the effect of cataract surgery on tear film stability on the ocular surface. Phacoemulsification was performed on 60 eyes under topical anesthesia by a clear corneal incision. Evaluation of the tear film included the OSDI, STT1, rose Bengal, and fluorescein staining. The TBUT and the TMH were measured noninvasively with the Keratograph 5 M (Oculus). Data were collected preoperatively, at 1 week, 1 month, and 3 months postoperatively. Postoperatively, all tests decreased significantly in the first week, before reaching the preoperative level after three months. The symptoms lasted up to three months.

The OCULUS Keratograph 5 M is a corneal topographer with a built-in real keratometer and a color camera optimized for external imaging. It is used for examining the meibomian glands, non-invasive TBUT, and the TMH measurement and evaluating the lipid layer. The Dry Eye Diagnostic System is a non-invasive system for dry eye analysis that provides quantitative data based on Artificial Intelligence (AI).

Sahu et al. [21] studied dry eye following phacoemulsification with the associated intra-operative risk factors in a prospective observational study. The study included 100 eyes of 100 patients without preoperative dry eye. The STT1 test, TMH, and LG staining of the cornea and conjunctiva were performed preoperatively and at 5 days, 10 days, 1 month, and 2 months postoperatively. The subjective symptoms were assessed using the dry eye questionnaire. The correlations between these values and the operating microscope light exposure time along with the cumulative dissipated energy (CDE) were analyzed. There was a significant deterioration of all dry eye test values postoperatively which started improving after 1 month. The preoperative levels were not achieved till 2 months postoperatively. The correlations of dry eye test values with the operating microscope light exposure time and CDE were not significant. Phacoemulsification surgery is capable of inducing dry eyes, The increased CDE can induce dry eyes even in eyes that were healthy preoperatively. In addition, intraoperative exposure to the microscopic light should be minimized.

In a prospective descriptive study, Kasetsuwan et al. [22] evaluated the incidence and severity pattern of dry eye after phacoemulsification in 92 uncomplicated cataract patients who were 18 years old or older. Dry eye incidence and pattern were analyzed at days 0, 7, 30, and 90 after phacoemulsification using OSDI, TBUT, Oxford ocular surface staining system, and STT1 test without anesthesia.

Staining, using the Oxford grading scheme, is the standard method used for the diagnosis of dry eye disease. The severity of staining was quantified using a chart comprising a series of panels, labeled A-E, of increasing severity. In each panel, fluorescein staining is represented by punctate dots. To grade the staining, comparisons were made between the panels and the appearance of staining on the exposed interpalpebral conjunctivas and corneas. The six Oxford scheme grades (0–5), which denote the severity of dry eye, were used to record the results. Specifically, the keratoconjunctival staining was rated mild (stage 0 or 1), moderate (stage 2 or 3), or severe (stage 4 or 5). In this study, 7 days after phacoemulsification, the incidence of dry eye was 9.8%. The severity of dry eye peaked seven days post-phacoemulsification and was measured by the OSDI questionnaire and all three clinical tests. Within thirty days and 3 months post-surgery, both the symptoms and signs showed rapid and gradual improvements, respectively.

Dodia et al. [23] evaluated dry eye associated with phacoemulsification surgery. The study included 272 patients undergone phacoemulsification cataract surgery assessed for TBUT and STTI. The patient demography percentage includes age group < 50 years (19.1), 50–65 years (54.4), and > 65 years (26.5), female (44.1) and male (56.9), urban (75.4) and rural (24.64), right eye (51.5) and left eye (48.5). The BUT and the STT1 at 45 post-operative days, were normal (84.6%), and dry eye (15.4%).

A significant change in TBUT and STT1 values was reported during the 1st and 7th postoperative days. Epi info version 3.5.1 was used to perform statistical analysis and an appropriate statistical test (Z-test) was used for analysis. Statistical *P* < 0. 05 was considered significant with a 95% confidence interval.

On the 45th postoperative day, 42 (15.4%) patients reported altered TBUT and STT1 values indicating dry eye. A statistically significant difference was observed between TBUT and STTI values on days 1 and 7 compared to pre-operative values. Maximum change in value was reported in both sexes above 65 years for 1st and 7th postoperative days. Similar findings were reported for TBUT value also.

Phacoemulsification cataract surgery with higher age and female sex was associated with a risk of dry eye. Significant percentage change of TBUT and STT1 values reported during 1st and 7th post-operative days compared to pre-operative status.

A study done by Sánchez et al. [24] to assess the intraobserver repeatability of automated, objective, and noninvasive measures obtained with the S390L Firefly WDR slit-lamp. The study included 50 eyes of patients with dry eye disease with a mean age of 55.06 ± 12.96 years. Three consecutively repeated measures of the following variables were obtained: first noninvasive break-up time (F-NIBUT), average noninvasive break-up time (A-NIBUT), tear meniscus height, tear meniscus area (TMA)., Intraobserver repeatability was estimated. It had been concluded that the S390L Firefly WDR slit-lamp has moderate intraobserver repeatability for F-NIBUT and A-NIBUT, suggesting that F-NIBUT and A-NIBUT are highly variable tests. The remaining variables show satisfactory intraobserver repeatability.

Comparing our results with other studies that mostly used conventional methods to assess the tear film, the most common findings in most studies were the statistically significant low values of the TBUT, and TMH, almost within one week after phacoemulsification. A variable gradual recovery over the next few weeks and months followed this deterioration [10, 11, 17–22]. In this current study, and another study done by Cetinkaya et al. [18], the MG and the LLT were not affected postoperatively. The reason behind these findings could be the damaged corneal nerves due to the corneal surgical incisions and to the surgical microscope light, and intensity. The goblet cells in the conjunctiva can also be damaged causing mucin deficiency, tear film insufficiency, and instability. During the surgery, the lid is protected by the surgical draping, hence it is not affected by the surgical incisions or the microscope.

Recovery time of the dry eye condition was variable between all the studies [17, 19–21]. We think the potential reasons for variation in recovery time probably depend on various factors such as the duration of the surgery, the length of the surgical incisions, corneal nerve, goblet cell damage, and the light and the intensity of the microscope light. The risk factors such as age, female sex, systemic diseases, systemic medications, psychiatric conditions, preexisting DED, meibomian gland dysfunction, preservatives in eye drops, surgery techniques, and lifestyle also affect the recovery time [12, 25].

## Conclusion

Phacoemulsification surgery can cause dry eye. Early diagnosis should be properly done and the condition should be managed properly to avoid complications. This study has been done to increase the awareness of ophthalmologists about post-cataract dry eye, its causes, and how to manage it. Uneventful cataract surgery can be followed by ocular discomfort, pain, and decreased visual acuity due to postoperative dry eye.

The patients should be instructed to avoid dusty, windy, and dry environments. Frequent blinking is advisable, Smoking and air conditioning should be avoided. Using a humidifier to dry indoor conditions and wearing sunglasses to protect the eye from the sun and the wind. The use of non -preservative eye lubricants 3–4 times daily for weeks or months may be required especially in chronic dry eye patients. However, in the long term, signs and symptoms of dry eye decrease, and dry eye test values return to preoperative values.

## Data Availability

No datasets were generated or analysed during the current study.

## Abbreviations

AI: Artificial intelligence
CDE: Cumulative Dissipated Energy
DED: Dry Eye Diseases
KC: Keratoconus
LLT: Lipid Layer Thickness
MG: Meibomian glands
nm: nanometer
NITBUT: Non-Invasive Tear Break Up Time
NITMH: Non Invasive Tear Meniscus Height
OSDI: Ocular Surface Disease Index questionnaire
TBUT: Tear Break Up Time
TMH: Tear Meniscus Height
STT1: Schirmer Tear Test 1
SPSS: Statistical Package for the Social Science
A-NIBUT: Average- NIBUT
F-NIBUT: First NIBUT
